# Reduction of Tetrachloroaurate(III) Ions With Bioligands: Role of the Thiol and Amine Functional Groups on the Structure and Optical Features of Gold Nanohybrid Systems

**DOI:** 10.3390/nano9091229

**Published:** 2019-08-29

**Authors:** Ditta Ungor, Imre Dékány, Edit Csapó

**Affiliations:** 1 Interdisciplinary Excellence Centre, Department of Physical Chemistry and Materials Science, University of Szeged, Rerrich B. square 1, H-6720 Szeged, Hungary; 2 MTA-SZTE Biomimetic Systems Research Group, Department of Medical Chemistry, University of Szeged, Dóm square 8, H-6720 Szeged, Hungary

**Keywords:** gold nanoparticles, gold nanoclusters, coordination polymer structure, amino acids, template-assisted synthesis, fluorescence, Au(I)-thiolate, gold nanohybrid materials

## Abstract

In this review, the presentation of the synthetic routes of plasmonic gold nanoparticles (Au NPs), fluorescent gold nanoclusters (Au NCs), as well as self-assembled Au-containing thiolated coordination polymers (Au CPs) was highlighted. We exclusively emphasize the gold products that are synthesized by the spontaneous interaction of tetrachloroaurate(III) ions (AuCl_4_¯) with bioligands using amine and thiolate derivatives, including mainly amino acids. The dominant role of the nature of the applied reducing molecules as well as the experimental conditions (concentration of the precursor metal ion, molar ratio of the AuCl_4_¯ ions and biomolecules; pH, temperature, etc.) of the syntheses on the size and structure-dependent optical properties of these gold nanohybrid materials have been summarized. While using the same reducing and stabilizing biomolecules, the main differences on the preparation conditions of Au NPs, Au NCs, and Au CPs have been interpreted and the reducing capabilities of various amino acids and thiolates have been compared. Moreover, various fabrication routes of thiol-stabilized plasmonic Au NPs, as well as fluorescent Au NCs and self-assembled Au CPs have been presented via the formation of *–*(Au(I)-SR)_n_*–* periodic structures as intermediates.

## 1. Introduction

Nowadays, the development of diverse nanostructured materials have a dominant role in several physical, chemical, medical, etc. fields from the electronics to the food industries [1,2]. The noble metal nanoparticles are extremely investigated nano-objects due to their electric, magnetic and unique morphology, size, and composition-dependent optical features [3,4]. This optical property originates from the so-called localized surface plasmon resonance (LSPR) phenomena, which results in the appearance of a characteristic plasmon band in the 400–800 nm range of the electromagnetic spectra [5,6]. In the last two-three decades, gold nanoparticles (Au NPs) have became increasingly the focus of interests in the material and medical sciences thanks to the advantageous physicochemical properties, such as large specific area, chemical inertness, and tunable optical particularity [7]. Several methods for fabrication of nano-sized Au NPs are known in the literature, including the physical (e.g., physical vapor deposition (PVD), microwave (MW) or ultraviolet (UV) radiation, ball milling or photoreductive routes, etc. [8,9]) and chemical approaches [3,4,10]. In the latter case, depending on the applied reducing and stabilizing agents (e.g., sodium borohydride [11,12], sodium citrate [13,14,15], surfactants [16,17], various amines [18], peptides [19,20], or biological organisms [21,22,23]), particles of different shapes and sizes can be produced. In the last decade, the sub-nanometer sized gold nanoclusters (Au NCs) have also became increasingly dominant. Beside the Au NPs, the Au NCs are also in the focus of researches. These ultra-small metal objects consist of only a few of few tens’ gold atoms, and generally the oxidation number of the Au is < 1 and Au–Au bonds can be found in the clusters. By the mentioned structure, the Au NCs show unique size-tunable photoluminescence (PL) due to the well-defined molecular structure and discrete electronic transitions [24,25,26]. The blue-emitting Au NCs usually only contain a few atoms, thus the emission band depends only on the number of atoms in the cluster and the PL lifetime occurs in the nanosecond range. Nevertheless, if the size of the Au NCs achieves the few-nanometer range (d ~1.5–2.0 nm), the characteristic emission band is detected in the orange and in the red visible region. In this case, the surface ligand effect and the oxidation state of the surface metal atoms both influence the location of the emission maximum and the PL lifetime reaches the microsecond range. The larger colloidal Au NPs (d ~2–10 nm) possess weak PL, which is regulated by the surface roughness and the grain size effect [27]. Based on the above-mentioned structure-depending optical features, the sub-nanometer Au NCs can potentially be used as optical probes for biosensing, bio-labelling, and bioimaging applications [24,26,27].

The biomedical applications (cancer therapy, diagnostics, and bioimaging, etc.) of nano-sized functionalized Au particles/clusters require biocompatible preparation routes with mild reaction conditions. Nowadays, the practical one-step “green” preparation protocols of several water-soluble Au NPs/NCs are extremely preferred [21,28,29,30]. During these processes, mainly the template-assisted preparation approaches are used, where dominant amines, like simple amino acids [31], peptides or proteins [32,33], dendrimers [34,35], and nucleotides [36,37,38,39], are applied, which have simultaneously a dual role as reducing and stabilizing ligand. The amines are a crucial class of the possible reducing agents, because they can be found in biological and chemical atmospheres. Main advantages of this relatively simple template-directed reduction technique are that no additional reducing agent is required and based on the well-defined structure of polypeptides and proteins uniform NPs/NCs with tunable optical features can be synthesized. Besides amines, the thiol group-containing molecules (e.g., thiolates) can coordinate and reduce the Au ions at the same time to form periodic *–*(Au(I)-SR)_n_*–* structures/complexes having partially reduced Au(I) ions, which are a well-known intermediates in the fabrication route of thiol-covered gold nanohybrid systems [40,41,42,43]. Several researches focus on the better understanding of the unknown structures of so-called atomically precise thiolate-protected Au NCs or the possible utilization of the thiolate-stabilized Au NPs/NCs [43,44,45]. In addition to the thiol-protected Au NPs/NCs, the study of the formation of Au-thiolate so-called “coordination polymer structure”, having Au^0^ or mostly Au(I) is in focus of interest. These coordination polymers (CPs) are inorganic-organic hybrid materials, which consist of periodic metal ions/atoms and ligand moieties and possess ordered structure. The self-assembly of this structure results in the formation of lamellar multilayers or helical structures with unique optical properties [41,46,47].

In recent work, we aim to provide an overview that is focused on the summary of the preparation routes, the unique structure, as well as the structure-dependent optical features of Au NPs, Au NCs, and Au CP structures that are synthesized by template-assisted synthesis exclusively using amines (mainly simple amino acids) and thiol-group containing molecules (e.g., thiolates) as possible reducing and stabilizing molecules. We mainly emphasize the formation of Au NPs, Au NCs, and Au CPs, which are fabricated by the direct interaction of tetrachloroaurate(III) ions (AuCl_4_¯) with amino acids and alkyl- and arylthiolates in the absence of other reducing agents. We clearly summarize the dominant effect of the metal ion concentration, the molar ratio of the precursor aurate ions and reducing bioligands, as well as the experimental conditions (e.g., reaction time, temperature, pH, etc.) on the tunable, structure-dependent optical properties (plasmonic or fluorescence) of the Au nano-objects.

## 2. Preparation of Amino Acid-Reduced Colloidal Au NPs Having Plasmonic Property

**Figure 1:**
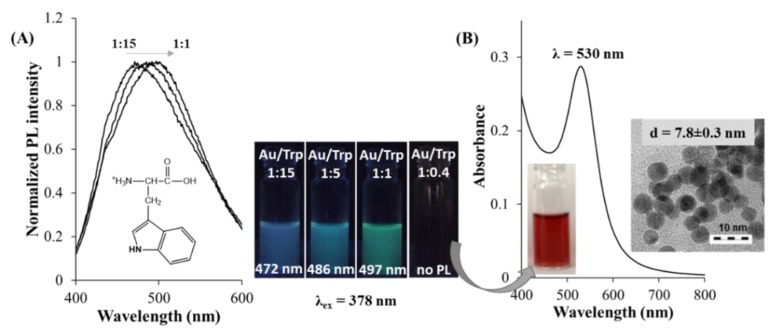
(**A**) The normalized fluorescence spectra (λ_ex_ = 378 nm) of L-tryptophan gold nanoclusters (Trp-Au NCs) with the photos of aqueous dispersions under UV-light. (**B**) Absorbance spectrum of L-tryptophan gold nanoparticles (Trp-Au NPs) with the HRTEM image. c(AuCl_4_¯) = 1.0 mM. Reproduced with permission from [71]. Elsevier, 2017.

There are several publications all around the world that describe the possible chemical synthesis routes of Au NPs in aqueous or in organic media. The well-known Brust method provides uniform alkyl or arylthiol-protected Au NPs (d = 1–5 nm) reduced by sodium borohydride (NaBH_4_) in toluene [11], while in aqueous medium the conventional method is the Turkevich process, which results in the formation of water-soluble Au NPs in the range of 5–50 nm reduced and stabilized by sodium citrate [13]. In the last decade, various other reduction and caption possibilities were examined, where bacteria and microorganisms [48,49], plant extracts [50,51], inorganic reagents [52], metal complexes [53,54], organic and physiological molecules [55,56], polymers [57,58], liposomes [59], etc. have been tested. Due to the biocompatible nature, easy accessibility, and remarkable reducing capabilities, the amino acids and their derivatives are used dominantly [60] to produce biocompatible noble metal NPs. As far as we know, to date, all the twenty naturally occurring amino acids were investigated. In 2002, Mandal et al. published firstly the formation of Au NPs having spherical shape and monodisperse size distribution (d = 25 nm) by spontaneous interaction of AuCl_4_^-^ with L-aspartic acid (Asp) under boiling condition while using AuCl_4_¯:Asp ca. 1:11 molar ratio [61]. Under the same experimental conditions, the synthesis was carried out with L-valine (Val) and L-lysine (Lys), but no reduction of AuCl_4_¯ was observed and during preparation, the role of the pH was not mentioned. Next year, the reduction capability of Lys was studied again [62], but Au NPs in the range of 6–7 nm could only be prepared at room temperature by the application of extra NaBH_4_ reductant as well. The hydrogen bonds between the surface-bound Lys molecules of the adjacent Au NPs was confirmed by NMR studies. Through the researches of Mandal, Selvakannan, and Sastry [63], L-tryptophan (Trp)-stabilized gold colloids was also efficiently fabricated. The synthesis was carried out at 50 °C while using AuCl_4_¯:Trp ca. 1:100 molar ratio. ^1^H NMR studies clearly indicated the indole-based polymerization of Trp, which contributed to the better understanding of the reduction process of Trp with AuCl_4_¯ forming Au NPs under mild reaction conditions without application of other harsh reducing agents like NaBH_4_. In 2005, Bhargava et al. summarized the successful fabrication of Au NPs by spontaneous interaction of potassium tetrabromoaurate(III) precursor (KAuBr_4_) with L-tyrosine (Tyr) and L-arginine (Arg) at room temperature while using ca. 1:4 metal ion to amino acid molar ratios under alkaline medium [64]. For Tyr-reduced Au NPs having 5–40 nm in size, a slightly polydisperse distribution and coagulations of the NPs were observed. The Arg-produced colloidal NPs have larger size than the average diameter of Tyr-reduced particles, but the size distribution showed much narrower shape. The cyclic voltammetry (CV) studies of Blanchard et al. provided important information regarding the reduction abilities of various amines, including amino acids L-glycine (Gly) and Trp, as well as the proposed reduction mechanism between metal ions and bioligands [65]. Presumably, the reduction of aurate ions occurs thanks to the electron transfer from amines to the metal ions resulting in Au atoms with zero oxidation state and finally the nucleation and growth steps eventuates the formation of NPs. This redox reaction results in the appearance of short chain amine oligomers, which is confirmed by NMR studies. Moreover, the oxidation potential of amines, which are used for the reduction of gold ions, has outstanding impact on the formation of Au NPs considering the reduction potential of AuCl_4_¯. Amines that have redox potential between the oxidation of Au^0^ to gold(I) and the reduction of tetrachloroaurate(III) to Au^0^ can be suitable used as reducing agents. L-Glutamic acid (Glu)-reduced Au colloids were also previously fabricated, having a particle size of d = 40 nm, but the synthesis was carried out under refluxing [66]. In 2010, the hydrothermal synthesis of the L-histidine (His)-reduced spherical Au NPs. The average diameter was 11.5 nm reported by Liu et al., where the AuCl_4_¯:His/1:2.5 molar ratio was used at 150 °C in alkaline (pH 11.50) medium [67]. The structural characterization of His-protected Au NPs supported that the terminal COO¯ group of His was not attached of the particle surface, while the imidazole as well as the amino groups were adsorbed on the Au surface. The construction of His-stabilized Au NPs did not occur at room temperature, but the hydrothermal conditions (e.g., high temperature and pressure) facilitate the formation of Au crystals. Besides the above-mentioned amino acids (Asp, Lys, Trp, Tyr, Glu, His), the reduction capabilities of L-aspartate (Asp), Gly, L-leucine (Leu), Lys, and L-serine (Ser) were also published by the work of Cai et al. in 2014 [68], but they used extra UV irradiation during the synthesis. The different Au NPs have diameters of 15–47 nm and the synthesis was carried out at pH 10.0 while using 1:10/AuCl_4_¯:amino acid molar ratios. Maruyama et al. studied the spontaneous interaction of each natural amino acids with aurate ions using high bioligand excess (metal ion to ligand ca. 1:100) at 80 °C, and they obtained that L-cysteine (Cys) and L-threonine (Thr) did not provide gold colloids. However, for L-methionine (Met) and L-phenylalanine (Phe), Au NPs were formed, but these colloids were easily precipitated. In 2014, L. Courrol and R. Almeida de Matos summarized their results in a book Chapter [69], where the formation of plasmonic Au colloids was confirmed by spontaneous interaction of aurate ions with Asp, Arg, Thr, Trp and Val using electromagnetic radiation (xenon lamp) at different pH using ca. 1:5 metal ion to amino acid molar ratios. However, the reduction capability of Trp was previously identified [70], but E. Csapó et al. clearly confirmed that the ratio of the precursor AuCl_4_¯ and the bioligand greatly influences the optical feature of the formed colloids [71]. Using AuCl_4_¯:Trp/1:0.4 molar ratio in alkaline medium (pH = 12.0), plasmonic Trp-Au NPs (λ_abs_ = 530 nm) were formed (Figure 1B). Based on the best of our belief, this work supported firstly that high ligand excess is no necessary for synthesizing Trp-reduced Au NPs at mild (37 °C) temperature. The presence of stable monodisperse Au NPs was confirmed by DLS (d_DLS_ = 8.8 ± 1.0 nm) and HRTEM (d_HRTEM_ = 7.8 ± 0.3 nm) studies. Moreover, depending on the applied molar ratios of the AuCl_4_¯:Trp, structure-dependent tunable optical property was also obtained. Namely, at acidic conditions (pH = 1.0), in the case of the mixing of Trp and AuCl_4_¯ solutions, the intensive yellow color of the solution changed to dark yellow after a few minutes. Below 1:1 ratio, unstable Au colloids was formed, but the application of molar ratio between AuCl_4_¯:Trp/1:1 and 1:15 resulted in luminescent products. The appearance of the emission peak depends of the ligand excess, namely the maximum value can be detected at λ_em_ = 497 nm (AuCl_4_¯:Trp/1:1), λ_em_ = 486 nm (AuCl_4_¯:Trp/1:5), and λ_em_ = 472 nm (AuCl_4_¯:Trp/1:15). The larger Trp amount causes the decrease of the PL intensities (Figure 1A). This characteristic PL originates from sub-nanometer sized Au nanoclusters (NCs). In the last 8–10 years, the Au NCs, which were synthesized by using template-assisted preparation routes, are in focus of extensive researches. A short summary of only the amino acid-reduced Au NCs is presented in the next chapter.

## 3. Synthetic Routes of Amino Acid-Reduced Fluorescent Au NCs

**Figure 2:**
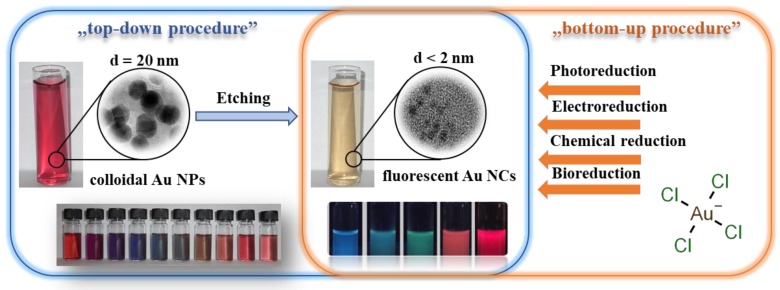
Preparation protocols of Au NCs by “top-down” and “bottom-up” approaches.

Several preparation protocols for Au NCs having sizes less than 2 nm have been established in the last two decades, including both the “top-down” and “bottom-up” approaches, as Figure 2 summarizes [25,72,73].

For the ”top-down” process, the larger colloidal particles undergo so-called “etching” in order to produce smaller clusters, while in case of “bottom-up” methods, the clusters are formed via a reduction of the precursor ions by assembling individual atoms one-by-one [34,74]. The ultra-facile, one-step synthetic processes are in focus of interest, where the execution of the reactions is very convenient, rapid, and mild, exempted from the application of harsh reducing agent, special ambience and media, and high pressure. However, numerous articles were published for the preparation of biocompatible Au NCs that were synthesized by template-assisted preparation protocols while using proteins and peptides [75,76], polymers [77], DNA [78], dendrimers [79], etc., but only a few publications present the possible applicability of simple amino acids as reducing and stabilizing agents.

**Figure 3:**
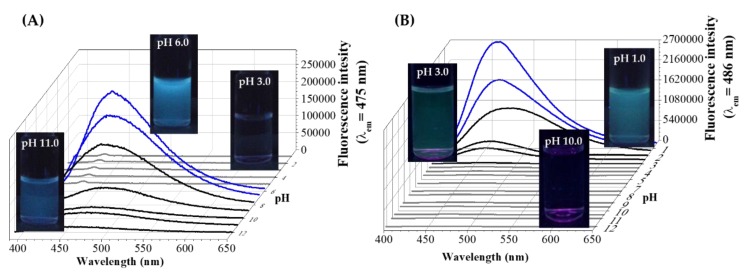
The photoluminescence (c) spectra as a function of the initial pH of the (**A**) AuCl_4_¯:His/1:30 and (**B**) AuCl_4_¯:Trp/1:5 systems with representative photos of the samples under UV-light. (λ_ex_ = 378 nm, c_Au-_ = 1.00 mM, T = 37 °C). Published in [71], Elsevier, 2017.

In this chapter, we clearly focus on the summary of the amino acid-directed fabrication of Au NCs having size-and structure-dependent intense PL features [80,81]. Table 1 clearly summarizes the experimental conditions of amino acid-reduced Au NCs and other Au-based nanohybrid structures. As it can be shown, His, Tyr, Pro, Trp, Cys, and Met amino acids were previously studied. Except for Cys and Met having thiol and thioether side chains, blue-emitting Au_3_-Au_10_ NCs can be synthesized by the spontaneous interaction of AuCl_4_¯ with His, Tyr, Pro, and Trp bioligands, depending on the temperature as well as on the ratio of reactant partners. In case of His, Au_10_ NCs with relatively high QY(%) are formed by using AuCl_4_¯:amino acid/1:30 molar ratio at room temperature [82]. As Table 1 summarizes, various research groups fabricated His-reduced Au_10_ NCs while using almost the same experimental conditions, where the His-protected Au NCs have been applied for glutathione detection and selective cancer cell imaging [83], while Liu et al. also successfully used the His-Au NCs as ultrasensitive iodide detector system [84]. It can be concluded that, at room temperature, the application of high ligand excess (30-fold excess) results the formation of His-stabilized blue-emitting NCs. Moreover, E. Csapó et al. clearly confirmed that the pH is also a decisive factor during the synthesis in the case of the His/AuCl_4_¯ system. However, Yang et al. [82] claimed that the emission intensity of the His-stabilized Au_10_ NCs was continually decreased with the increase of pH (from pH = 1.0 to 13.0) and the extreme acidic condition (pH = 1–2) is optimal for these NCs. In contrast with their results, E. Csapó et al. found that (Figure 3A), if the pH is smaller than pH = 5.0 no emission could be detected, but a characteristic emission peak with continually decreasing intensity to pH = 12.0 was evolved at 475 nm at above pH > 6 [71]. The emission maximum values show an interesting correlation with the concentration distribution curves of His. Namely, the emission maximum can be observed in that pH, where the deprotonation of the imidazolium moiety of His eventuates (pK_a_ = 6.04) [85].

Most probably, the primary coordination of the gold ions to the His occurs via the imidazole-*N* atoms and this aromatic group plays a dominant role in the formation of the fluorescent Au products. Furthermore, it was found that, through the decrease in the concentration of the AuCl_4_¯ ions from c_Au_ = 2.50 mM to c_Au_ = 1.00 mM, instead of clusters, the presence of blue-emitting polynuclear Au(I) complexes having a well-ordered structure is certifiable by several analytical methods [71].

For Tyr, no high ligand excess is necessary, but at room temperature, the spontaneous interaction of the Tyr with AuCl_4_¯ ions does not result in the fabrication of Tyr-reduced Au NCs. At higher concentrations (c_Au_ = 2.50 mM), the lower temperature is enough (37 °C), but the boiling condition is essential as the concentration decreases (c_Au_ = 0.07 mM). In the case of Pro, which does not contain an aromatic group in the side chain, the use of extreme high ligand excess (more 100-fold excess) and boiling can result in the production of Au NCs having a few gold atoms. For Trp, the 37 °C and the 100 °C is optimal for the synthesis using from 1:1 to 1:5 AuCl_4_¯:Trp molar ratio at acidic condition, as in Figure 3B, and the previously mentioned tunable optical feature was found, depending on the reactants ratio, which was summarized in chapter 2 in Figure 1A.

In case of Met and Cys amino acids, which have thiol and thioether moieties in the side chain, the characteristic PL emission band was detected at higher (in the yellow and orange regions between 520–630 nm) wavelengths. However, for Met, the formation of Au NCs having Au^0^ cores was confirmed, but the pH and the temperature were extremely changed during the two-step preparation route. The spontaneous interaction of thiol-group containing Cys with AuCl_4_¯ does not result in clusters. Instead, a periodic Au(I) CPs was identified at pH = 3.0 by Söptei et al. measurements [94]. This nanohybrid system has a multilayered construction with 1.3 nm of distance and show characteristic fluorescence thanks to the (-S-Au(I)-S-Au(I)-S-)_n_ cyclical structure, which was verified by previously published similar Au(I)-thiolate systems [95,96]. In conclusion, the application of simple amino acids having aromatic groups (imidazole, indole, benzene) in the side chains dominantly results in the formation of fluorescent Au NCs. In contrast with the larger polypeptides or proteins, which mainly form red-emitting NCs [97], by the utilization of amino acids as reducing agents, only blue-emitting sub-nanometer sized NCs that consist of a few atoms can be synthesized. At lower synthesis temperature (e.g., room temperature), the application of higher ligand excess (ca. 30-fold excess) is advantageous, but, by increasing of the temperature (~40–50 °C), the use of high ligand excess can be reduced. The bioligands like Cys or Cys-containing small peptides, do not produce fluorescent NCs having Au^0^, but the formation of partially reduced –(Au(I)-SR)_n_– periodic structures is especially preferred. The preparation possibilities of –(Au(I)-SR)_n_– structures as well as the synthesis routes of thiolate-stabilized Au NPs/NCs and CPs through the –(Au(I)-SR)_n_– are summarized in the next chapter.

## 4. Fabrication Protocols of Thiolate-Protected Au Nanohybrid Systems

**Figure 4:**
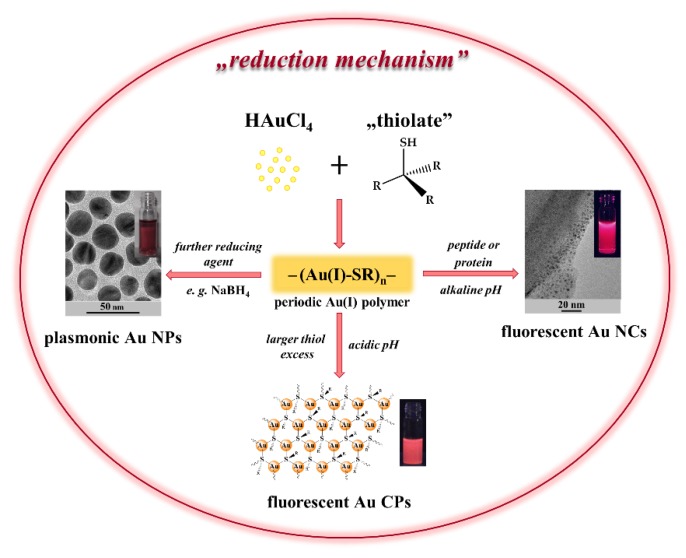
Schematic illustration on the formation mechanisms of different Au nanohybrid systems via interaction of tetrachloroaurate(III) ions with thiolate ligands.

Various publications can be found in the literature, relating to Au nanostructures that are synthesized by the interaction of AuCl_4_¯ ions with thiolate molecules as Cys amino acid, peptides having Cys residue or alkyl- and arylthiolates. Depending on the applied fabrication parameters (e.g., chemical structure of the reducing ligand, temperature, molar ratio, pH), decisively three different types of gold-thiol nanohybrid systems, such as plasmonic Au NPs or fluorescent Au CPs and Au NCs, as in Figure 4, can be fabricated. Nevertheless, the presence of similar bond (e.g., covalent bond) between the gold and the sulphur atom(s) of the applied bioligands was confirmed for all the nanostructures.

As mentioned in chapter (2.), one of the most commonly used synthesis is the two-phase Brust method for the formation of thiol-protected plasmonic Au NPs [11]. To simplify this method, C. K. Yee et al. developed a protocol, where only tetrahydrofuran was applied as individual solvent [98]. In both methods, several functionalized colloidal particles have been synthesized, which are functionalized by different alkyl- or arylthiols. The size of these Au NPs can be tuned by the molar ratio of the AuCl_4_¯:thiol-containing molecule, but the one-phase synthesis eventuates larger plasmonic particles [99]. For the exact understanding of these syntheses, Perala and Kumar presented a new synthetic route [100], where the formation of the particle consists of a two-step reduction mechanism, as demonstrated by the Equations (1) and (2).

AuCl_4_¯ + 4RSH → –(Au(I)-SR)_n_– + RSSR + 4Cl¯ + 3H^+^ (1)

–(Au(I)-SR)_n_– + BH_4_¯ + RSH + RSSR → Au_x_(SR)_y_ (2)

Based on the proposed mechanism, the first two equivalents alkyl- or arylthiol partially reduces the AuCl_4_¯ ions to Au(I), while next two equivalents involve in the formation of a periodic –(Au(I)-SR)_n_– polymer [101]. The final Au(I) → Au^0^ reduction is carried out by a borohydride salt, which results in the formation of Au_x_(SR)_y_. After reduction, the nucleation, as well as the crystal growth and the particle functionalization, are simultaneously occurred.

As it can be seen, the formation of thiol-protected Au nanohybrid systems occurs through the appearance of a periodic –(Au(I)-SR)_n_– polymer structure. These periodic polymers can simply be further transformed into new gold-containing products having different structure and optical properties (Figure 4). (i) On one hand, the utilization of strong reducing agents (e.g., NaBH_4_) results in colloidal Au NPs having plasmonic feature; (ii) by the application of a large excess of bioligand having thiol group in the side chain, such as Cys amino acid [40] or glutathione (GSH) tripeptide [102], the formation of Au CPs structures, including self-assembly structure at acidic conditions, is preferred; and, (iii) for the presence of peptide or protein reducing agents excess, fluorescent Au NCs can be synthesized.

**Figure 5:**
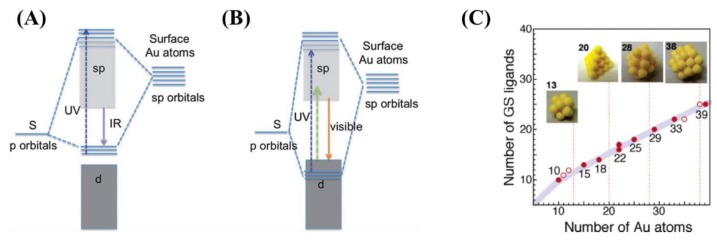
The scheme of the sp and d transitions in case of the (**A**) NIR- and (**B**) visible-emitting thiolate-protected Au nanohybrid systems. Reproduced with permission from [81], RSC, 2012. (**C**) The relationship between the Au atoms and glutathione (GSH) ligands in the most dominant (●) and secondary (ο) products. Reproduced with permission from [105], ACS, 2005.

These mentioned nanostructures (especially the NCs and CPs) possess intense structure-dependent PL mostly in the orange and red visible or the near infrared (NIR) region. The hybrid electronic states are formed between the sulphur atoms of the ligands and the gold atoms, which results in the emission from the sp to d band transitions [81]. These hybrid bands are below the d band states of Au(I) ions and the excitation wavelength-dependent fluorescence lifetime suggests that the triplet and singlet states are degenerated. In contrast, the hybrid orbitals are above the d band states of gold in case of NIR emission and the microsecond fluorescence lifetime refers to the strong involvement of the Au(I)-S charge transfer in the emission process (Figure 5A,B). In this chapter, the preparation protocols of Au CPs as well as the Au NCs systems were mainly interpreted.

In the case of earlier reports, the pH was not really regulated in the initial stage of the “green” synthesis as well as quite small GSH, Cys, or another thiolates excess was applied. Whereupon, NaBH_4_ was usually necessary to supplement the reduction process. As a result of the simple reaction of GSH and HAuCl_4_, T. G. Schaaff and R. L. Whetten identified three different GSH-Au(I) polymers. The AuCl_4_¯:GSH/1:3 molar ratio, ca. 0.3 mM of HAuCl_4_ concentration and ten-fold excess of NaBH_4_ in methanol:water solvent mixture were applied at room temperature, which prevent the polymer from the uncontrolled reduction [103]. The separation of the dark brown products was carried out by polyacrylamide gel filtration (PAGE) and the average sizes of the polymers were 4.3, 5.6, and 8.2 kDa. These nanohybrid systems show strong structure-dependent optical properties in the NIR, visible and UV-region, while the unseparated mixture nor. Y. Negeshi et al. also investigated the effect of the GSH and homo-GSH on the HAuCl_4_ in two articles. In contrast to the previous result, AuCl_4_¯:GSH/1:4 molar ratio and 4 mM of tetrachloroaurate(III) concentration were adjusted with a large excess of NaBH_4_ at 0 °C [104,105]. The identification of the dark-brown powder was accomplished after the PAGE and ultracentrifugation. The nine different Au(I)-polymer structures were recognized by Electrospray Ionization Mass Spectrometry (ESI-MS), optical absorption, and PL spectroscopy (Figure 5C).

This article presented firstly that, the smaller structures have rather polymeric properties such as the larger emission wavelength and larger binding energy (Au 4f_7/2_ ~85 eV), which refers to the decisive presence of Au(I). On the other hand, the systems having larger sizes show cluster-like characteristics with higher emission energy and the binding energy was detected at 84–85 eV. Thereby, the relationship was clearly pointed out between the size, the structure, and the optical behavior of the Au nanohybrid systems.

Neglecting of further reducing agents, R. E. Bachman et al. applied a phenylthiolate to synthesize a fluorescent and self-assembly gold(I) polymeric structure via decomposition of isonitrilegold(I) complex [106]. For the formation of supramolecular system, the dimer units aggregated in an antiparallel fashion at 255 °C, which can be described as a “crinkled tape” motif. It has strong PL in the red region at λ_em_ = 660 nm due to the weak aurophilic interaction in the supramolecular system. I. Odriozola et al. also examined the direct interaction of GSH and AuCl_4_¯ while using 1:3/gold: ligand molar ratio without the utilization of any further reducing chemicals at room temperature [107]. In their publication, the sol-gel transition was demonstrated, by which the prominent role of the pH on the gold(I)-thiolate structure was discussed. The possible chemical structures of the sol and the gel state were also suggested. H. Nie et al. 3-mercaptopropionic acid, thioglycolic acid, 1-thiogliycerol, and GSH were used to synthesize Au CPs with metal ion: ligand/1:1 stoichiometry [108]. As several CPs have great UV-Vis absorptions that originate from the ligand to metal and the metal-centered charge transfers, thereby the prepared nanohybrids are suitable for the *in-situ* checking the self-assembly of thiol-Au(I) CPs. The synergic effects of the weak interactions were identified with applying different analytical methods (e.g., time-resolved UV-Vis spectrophotometry, HRTEM, X-ray diffraction/XRD, and X-ray photoelectron spectroscopy/XPS). Consequently, it has been proved that the H-bonding, aurophilic and static interactions, and coordination bonding facilitate the evolution of the order structure for Au(I) CPs. C. Lavenn et al. also used phenylthiolate to prepare Au CPs by the development of a hydrothermal method at 120 °C [41]. The formed double helical Au CPs are also stabilized by C-H·π and aurophilic bonds. The product has red emission (λ_em_ = 684 nm) and great quantum yield (~5%). Furthermore, a thermally induced crystallization was presented in solid-state, which rarely occurred in gold(I) polymers. A. T. Royappa et al. applied two different water soluble ethanol-based thiolate molecules to produce of Au(I) CPs while using AuCl_4_¯:thiol/1:3 molar ratios [109]. The synthesis had a nearly quantitative yield and an amorphous colored gel-like solid was identified as periodic coordination polymer structure, which contains significant aurophilic interactions between the gold atoms.

**Figure 6:**
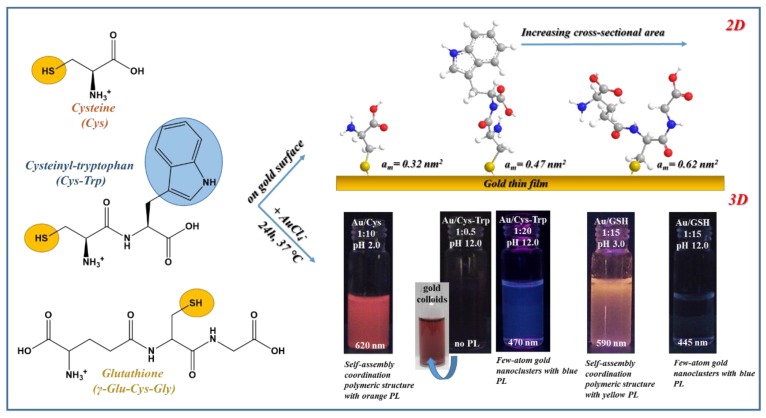
Schematic illustration of the binding of Cys and Cys-containing peptides on gold surface with the corresponding cross-sectional area (above) and the formation of Cys-, Cys-Trp-, and GSH-reduced Au NPs, Au NCs, and Au CPs by spontaneous interaction of the mentioned molecules with AuCl_4_¯ with some representative images. Published in [93], Elsevier, 2016.

Besides the previously mentioned, mainly thiolate-based Au CPs, the possible use of biocompatible amino acid Cys is in the focus of interest, especially in the last five years. P. S. Capellari et al. synthesized of ~0.6 nm ultra-small Cys-capped plasmonic Au NPs by precise growth controlling in mild conditions while using pH switching [110]. For understanding the formation mechanism, both acidic and alkaline conditions were examined. The applied molar ratio was ca. AuCl_4_¯:Cys/1:1 with 5 mM of HAuCl_4_ concentration at room temperature. Thanks to their experiments, two very stable polymeric gold(I)-thiolate structure were discerned at the two edges of the pH range and a rather reactive pH interval was identified between 4 < pH < 9. Based on several X-ray analytical methods, the structure of the Cys-Au(I) polymer show strong pH-dependence due to the zwitterionic nature of the Cys. The reactive state was suitable for controlled synthesizing of the plasmonic particle from the stable polymeric structures by pH switching and the adding of NaBH_4_. For the structural characterization, B. Söptei et al. examined the pale-yellow solid powder by small- and wide-angle X-ray scattering (SWAXS), which was formed by the direct reduction process between the Cys and AuCl_4_¯. For the preparation, AuCl_4_¯:Cys/1:10 molar ratio with 5 mM of gold concentration and three different temperature were tested without any regulation of the pH [94]. In their publication, a periodic lamellar structure was presented based on the SWAXS measurements, where the average distance of the lamellas was 1.3 nm. Beside these, the primary coordination bonds were defined by FT-IR spectroscopy. In the IR spectrum of the lamellar structure, the band corresponding to the S-H vibrations was disappeared, while a band was observed at the C = O stretching vibrations. These referred to the Au-S bond in the polymer structure, which were stabilized by strong H-bonds and electrostatic interactions due to the zwitterionic behavior of the Cys amino acid. E. Csapó et al. also examined the spontaneous reaction of the Cys and two cysteine-containing peptides with AuCl_4_¯ ions while using 1.0 mM of gold concentration at 37 °C in aqueous medium [93]. Depending on the applied pH, the molar ratios and the chemical structure of the Cys and Cys-containing peptides (Cys-Trp, GSH), diverse nanohybrid systems were formed, as in Figure 6. For understanding the ligand-dependent structures of these produced systems, two-dimensional (2D) techniques (surface plasmon resonance and quartz crystal microbalance) were additionally applied. In both cases, orange-emitting products (λ_em_ = 620 and 590 nm) were confirmed while using AuCl_4_¯:Cys/1:10 and AuCl_4_¯:GSH/1:15 ratios, respectively. Under acidic conditions (pH 3.0), the coordination polymers were identified and the lamellar architecture with 1.3 nm distance of the Cys-Au(I) CPs is also certified by XRD. Nevertheless, the ordered structure of GSH-Au(I) CPs was not verified, probably for the larger space-filling of the side chain. Under basic conditions, the orange emission was not observed in the GSH-Au system, but a new blue emission band was involved at 445 nm. The XPS studies of this system supposed the formation of ultra-small Au^0^ clusters. In contrast of Cys, the redox potential of GSH shows a strong pH-dependent property, thus the tripeptide has stronger reduction capability against the Au(III) ions. Next to the redox feature of the GSH, the hydrolytic process of the aurate(III) ions also influences the structure of final gold products. The presence of AuCl_4_¯ is dominant between pH = 1–3, but, at basic conditions, the appearance of various hydroxo species (e.g., AuCl(OH)_3_¯ or Au(OH)_4_¯) is exclusive.

The amine and thiol-containing dipeptide, named cysteinyl-tryptophan (Cys-Trp), showed mainly amino acid behavior against the AuCl_4_¯. Depending on the applied ligand amount, the optical properties of the formed gold systems can be tuned. With a small quantity of the Cys-Trp (1:0.5/AuCl_4_¯:ligand ratio) under basic conditions, plasmonic Au NPs were synthesized with ca. 8–9 nm. In contrast, while using 20-fold dipeptide excess two-coordinated Au(I)-complexes with blue emission (λ_em_ = 470 nm) were identified by the MS techniques. The supramolecular self-assembly of these complexes was not observed, presumably also due to the large size of the ligand. The thioether Met amino acid was used for synthesizing Au NCs by H. H. Deng and co-workers [92]. For the preparation of Met-Au NCs, extreme large Met excess and a two-step thermostated reaction were applied in alkaline medium. The identified cluster shows yellow emission at 530 nm and the quantum yield was 2.9% with two dominant fluorescence lifetimes (181 ns and 1.6 µs). The XPS spectrum suggested that the cluster decisively built up from Au^0^. Based on the FT-IR studies, the functional groups of –NH_2_ and –COOH take part in the formation of the coordinative bonds on the cluster surface, but not on the sulphur atom.

As it can be seen, the application of simple (bio)thiolates as simultaneous reducing and stabilizing agent results Au(I)-containing periodic polymer products in most cases. For the synthesis of thiol-reduced Au NCs, either other reducing agents (e.g., borohydride salts) or proteins are usually required. Forasmuch, this article is limited to detailed descriptions of the direct interaction between small amines and thiols, only the brief introduction of the mechanism of the protein-tetrachloroaurate(III) reaction is as follows, because the peptides can be considered as large-sized biocompatible thiolates and amines. Several articles can be found on the syntheses of protein-stabilized Au NCs while using the BSA [111,112,113], HSA [114,115], LYZ [116,117,118,119,120], trypsin [121], pepsin [122], or immunoglobulin [76]. The typically red-emitting cluster synthesis is carried out under basic conditions (~pH 12) and 10–20-fold protein excess is applied at ca. 40 °C for 24 h. The purification can be done by dialysis or PAGE techniques. The synthesized Au_25_ NCs contain a core having icosahedral Au_13_, which are covered by an Au_22_ shell and they are stabilized by 18 thiolate ligands based on the X-ray crystallographic analysis [123]. Nevertheless, the general accepted mechanism of the cluster formation is the follows. The complete reduction of the Au(III) to Au^0^ also occurred via a precious presented two-steps process. The primary Au(III) → Au(I) progress occurs along the side chain of Trp and Tyr residues. Following a “chain migration”, the gold(I) ions are coordinated by the sulphur-containing molecules, where the further reduction is realized by the nearby and suitable amino acids. On one hand, the used extreme basic conditions serve to improve the reduction capability of the Tyr and Trp amino acids. On the other hand, the unfolding of the protein chain is also contributed by applying of alkaline medium, which facilitates easier migration of the partially reduced metal ions along the chain. Based on the above considerations, the presence of the adequate Tyr and Trp beside the thiol-containing amino acids is definitely an important criterion for the success of Au NCs syntheses [124,125,126]. It can be regarded that the proteins are a great bridge between the biocompatible amine and thiolate ligands.

## 5. Conclusions

The gold nanoparticles, the ultra-small Au nanoclusters consisting a few or few tens of gold atoms, and the Au-containing self-assembled coordination polymers are in focus of extensive researches thanks to their several excellent properties. Due to the low toxicity as well as their unique, structure-dependent optical feature, they can be used in several fields of medical applications, like as the controlled drug delivery, cancer treatment, fluorescence imaging, diagnostic, and sensing. One of the most important requirements in these medical utilizations is the biocompatibility and the synthesis of these nanostructures under mild reaction conditions in aqueous medium using biocompatible capping agents and avoiding the harsh reducing agents or organic solvents, etc. Based on these expectations, in this review we decisively focused on the short summary of the possible synthetic routes of the formation of colloidal Au NPs, Au NCs, and Au CPs via template-assisted preparation protocol while using amino acids and thiolates as reducing and stabilizing molecules.

For amino acids we can conclude that, almost all amino acids, except Cys, are able to reduce the precursor AuCl_4_¯ ions at mostly high temperature (T = 50–100 °C), and the formation of stable colloidal Au NPs is preferred. Besides the higher temperature, the high pressure, as well as the extra conditions, like alkaline medium, the high ligand excess or the application of UV light further facilitate the appearance of Au NPs, having sizes larger than 2 nm. In the case of fluorescent amino acids-reduced Au NCs, only the possible utilization of His, Trp, Pro, and Tyr having aromatic residues in the side chain was confirmed to date. At lower synthesis temperature (e.g., room temperature), the application of higher ligand excess (ca. 30-fold excess) is advantageous, but, by increasing of the temperature (~40–50 °C), the use of high ligand excess can be reduced.

The Cys or Cys-containing peptides do not produce fluorescent NCs, but the formation of Au(I)-containing polymers having an ordered structure is especially preferred. The preparation possibilities of these structures through the periodic –(Au(I)-SR)n– as well as the characteristic features of thiolate-stabilized Au NPs/NCs and CPs were also summarized. As presented, the detailed examination of the relationship between the reaction conditions and the optical/structural features of the formed Au-containing nanohybrid systems is extremely important for future applications. Due to the effective PL quenching of Au NCs and Au CPs or the LSPR phenomena of Au NPs, these nanostructures are potential candidates for Photodynamic therapy (PDT), Photothermal therapy (PTT), and X-ray imaging. Moreover, these nanosized noble metal-based nanohybrid structures play a decisive role as possible nanosized controlled drug delivery systems in pharmaceutical applications. Moreover, the sub-nanometer sized fluorescent NCs are excellent nanosensors for rapid and selective detection of essential (Fe(III), Cu(II)) and toxic (Hg(II), Cd(II)) metal ions, anions (e.g., CN^−^), or biological molecules (e.g., glucose, folic acid, glutathione, toxins, drugs, etc.)

## Figures and Tables

**Table 1:** Experimental conditions of amino acid-reduced Au NCs and Au nanostructures.

Amino Acid	c_AuCl4_ (mM)	AuCl_4_¯:Amino Acid Ratio	T (°C)	Product	λ_ex_ (nm)	λ_em_ (nm) QY (%)	Ref.
His	2.50	1:30	25	Au_10_ NCs	386	490 (8.78%)	[82]
His	2.50	1:30	25	Au_10_–Au_14_ NCs	370	475 (*no inf*.)	[86]
His	2.50	1:30	25	Au NCs *	386	475 (*no inf*.)	[83]
His	2.50	1:30	25	Au NCs *	365	450 (4.60%)	[84]
His	2.50	1:45	25	Au NCs *	386	498 (8.96%)	[87]
His	1.00	1:30	37	Au(I)-His CP	378	475 (3.60%)	[71]
Tyr	2.50	1:1.8	37	Au NCs *	385	470 (2.50%)	[88]
Tyr	0.07	1:0.76	100	Au_10_ NCs	383	498 (1.68%)	[89]
Pro	2.40	1:830	100	Au_7_ NCs	365	440 (2.94%)	[90]
Trp	0.43	1:2.7	100	Au_8_ NCs	365	450 (*no inf*.)	[91]
Trp	0.50	1:1	37	Au_3_–Au_6_ NCs	378	497 (1.10%)	[71]
	0.50	1:5	37	Au_3_–Au_6_ NCs	378	486 (1.30%)	[71]
	0.50	1:15	37	Au_3_–Au_6_ NCs	378	472 (1.70%)	[71]
Met	4.06	1:20	37	Au NCs *	420	530 (2.80%)	[92]
Cys	1.00	1:10	37	Au(I)-Cys CP	395	620 (*no inf*.)	[93]
Cys	5.00	1:10	25	Au(I)-Cys CP	365	630 (1.10%)	[94]

* no data are available for the number of gold atoms in the clusters.
